# Molecular Mechanisms of Resistance to Immune Checkpoint Inhibitors in Melanoma Treatment: An Update

**DOI:** 10.3390/biomedicines9070835

**Published:** 2021-07-18

**Authors:** Sonja Vukadin, Farah Khaznadar, Tomislav Kizivat, Aleksandar Vcev, Martina Smolic

**Affiliations:** 1 Department of Pharmacology and Biochemistry, Faculty of Dental Medicine and Health Osijek, Josip Juraj Strossmayer University of Osijek, 31000 Osijek, Croatia; sonja.vukadin@fdmz.hr (S.V.); farah.khaznadar.8@gmail.com (F.K.); 2 Department of Pharmacology, Faculty of Medicine, Josip Juraj Strossmayer University of Osijek, 31000 Osijek, Croatia; 3 Clinical Institute of Nuclear Medicine and Radiation Protection, University Hospital Osijek, 31000 Osijek, Croatia; tomislavkizivat@gmail.com; 4 Department of Nuclear Medicine and Oncology, Faculty of Medicine Osijek, Josip Juraj Strossmayer University of Osijek, 31000 Osijek, Croatia; 5 Department of Pathophysiology, Physiology and Immunology, Faculty of Dental Medicine and Health Osijek, Josip Juraj Strossmayer University of Osijek, 31000 Osijek, Croatia; avcev@fdmz.hr; 6 Department of Pathophysiology, Faculty of Medicine Osijek, Josip Juraj Strossmayer University of Osijek, 31000 Osijek, Croatia; 7 Department of Internal Medicine, University Hospital Osijek, 31000 Osijek, Croatia

**Keywords:** melanoma, drug resistance, immune check-point inhibitors, immunotherapy, ipilimumab, pembrolizumab, nivolumab

## Abstract

Over the past decade, immune checkpoint inhibitors (ICI) have revolutionized the treatment of advanced melanoma and ensured significant improvement in overall survival versus chemotherapy. ICI or targeted therapy are now the first line treatment in advanced melanoma, depending on the tumor v-raf murine sarcoma viral oncogene homolog B1 (BRAF) mutational status. While these new approaches have changed the outcomes for many patients, a significant proportion of them still experience lack of response, known as primary resistance. Mechanisms of primary drug resistance are not fully elucidated. However, many alterations have been found in ICI-resistant melanomas and possibly contribute to that outcome. Furthermore, some tumors which initially responded to ICI treatment ultimately developed mechanisms of acquired resistance and subsequent tumor progression. In this review, we give an overview of tumor primary and acquired resistance mechanisms to ICI and discuss future perspectives with regards to new molecular targets and combinatorial therapies.

## 1. Introduction

Melanoma is a skin malignancy caused by uncontrolled growth of abnormal melanocytes. Although an uncommon skin malignancy, it is well known for its very poor survival rate [1]. The incidence rate is rising worldwide with age, and it is mostly diagnosed in the population aged between 70 and 80 [1]. This has implications on the choice of a drug regimen. Diagnosis of melanoma at an early stage is still crucial for the most favorable outcome, while in metastatic disease less than 10% of patients completely recover [2].

Melanoma responds poorly to chemotherapy and interferon therapy, but relatively recent findings on melanoma biology at the molecular level have resulted in the development of new treatment options: immune checkpoint inhibitors (ICI) and targeted therapeutics [3]. Blocking immune checkpoints reactivates T cells and enables their cytotoxic effect on tumor cells [4]. Melanoma was the first FDA-approved indication for the use of cytotoxic T-lymphocyte-associated protein 4 (CTLA-4) inhibitor, ipilimumab. Only 3 years later an anti-programmed death ligand 1 (anti-PD-L1) monoclonal antibody, pembrolizumab, gained approval for the use in advanced melanoma [5]. In the same year, the third immune checkpoint inhibitor (ICI) nivolumab, an IgG1 monoclonal antibody against programmed cell death protein 1 (PD-1), was approved in some parts of the world [6].

Unfortunately, ipilimumab treatment was shown to be effective in only 22% of melanoma patients after 5–10 years of therapy, while successful treatment with PD-1 inhibitors was achieved in 40–45% of patients with melanoma. Treatment with combination ICIs (PD-1 inhibitor and CTLA-4 inhibitor) resulted in a higher response in comparison to single agents, but with the cost of higher toxicity. Therefore, the risks can outweigh the benefits.

Undoubtedly, the introduction of ICI to melanoma treatment has been revolutionary, considering that previously patients were limited to chemotherapy with dacarbazine, which had a modest efficacy and included a significant risk of toxic side effects. A change in median survival improved from 6–10 months to 24 months or more [7]. Furthermore, a 5-year overall survival rate of 52% with combination therapy of ipilimumab and nivolumab has been reported [8]. However, although ICI improved survival in metastatic melanoma in a significant proportion of patients, 40–65% of patients receiving PD-1 inhibitors and more than 70% of patients with CTLA-4 inhibitors did not respond due to primary resistance mechanisms [9]. Furthermore, some cases which had a good clinical response at first, eventually developed drug resistance and tumor progression (acquired resistance). In almost one third of patients with metastatic melanoma who initially responded to ICI therapy, there was melanoma progression within 3 years [10].

Numerous mechanisms that underlie resistance to ICI have been elucidated. However, many are not well understood. Given that efficient ICI therapy requires proper function and tumor microenvironment (TME) infiltration by effector T cells, tumor resistance to ICI therapy is noted in circumstances where malignant cells impair T cells at different stages of their maturation or effector function [9]. Understanding the mechanisms and conditions under which melanoma cells evade immune surveillance by T cell suppression would allow the development of specific biomarkers and the selection of responders to ICI. This would also open additional avenues for the research of new therapeutic agents to block the pathways responsible for the failure of ICI treatment [9,11].

In this paper, we give an overview of immune checkpoint proteins and their role in immune response, and discuss molecular mechanisms of primary and acquired melanoma resistance to ICI with proposed strategies to overcome them. We also discuss the future directions in melanoma treatment. 

## 2. Immune Checkpoint Proteins and Pathways

Immune checkpoints are inhibitory membrane receptors expressed on activated T cells. Engagement with their specific ligands leads to T cell suppression in order to prevent their action against host tissue, which is a normal regulatory process [11]. Understanding how immune checkpoints function elucidated many aspects of autoimmune and chronic inflammatory diseases [12,13]. In the current review, we focus on checkpoint protein function in the context of melanoma. To date, in-depth characterization of two of the immune checkpoint proteins and their regulatory effects yielded a design of drugs which were approved for treatment of advanced melanoma, CTLA-4 and PD-1 inhibitory proteins and PD-L1, a ligand of PD-1.

A monoclonal antibody to immune checkpoint CTLA-4 called ipilimumab, was the first ICI which gained approval to be used in the treatment of advanced melanoma [14,15]. CTLA-4 activation has a role in T cell suppression upon its stimulation by antigen. T cell activation besides engagement between T cell receptor (TCR) and major histocompatibility complex/ antigen (MHC/Ag) complex on antigen-presenting cells (APCs) requires at least one more stimulatory signal [16]. APCs express B7 proteins on their cell surface, which can bind to both CTLA-4 and CD28 on T cells. While CD28 activation produces co-stimulatory effects on T cells promoting their proliferation and cytokine release, CTLA-4 acts as a T cell inhibitor, promoting anergy or apoptosis. Therefore, those two receptors compete for a shared ligand, with CTLA-4 being dominant due to its higher affinity to B7. The net effect on T cells depends on the ratio of CD28-B7 and CTLA-4-B7 complexes [16]. The inhibitory role of CTLA-4 is important for the prevention of an exaggerated T cell response. Its downregulation in a mouse model has been shown to cause uncontrolled lymphoproliferation and tissue destruction [17]. Similarly, in humans it has been linked to various autoimmune and lymphoproliferative conditions [18]. CTLA-4 expression is upregulated by CD28-B7 complex formation, among other signals [19]. Some solid tumors, including melanoma, exploit this CTLA-4-mediated T cell inhibition to create a favorable environment for their progression. 

Another important factor in the immunomodulatory process are T regulatory cells (T_reg_). T_reg_ use CTLA-4 to suppress effector T cells and in that way sustain tolerance [20]. T_reg_ are also exploited by the malignant cells, which use them as a shield against cytotoxic T cells [21]. T_reg_ release immunosuppressive cytokines, transforming growth factor-β (TGF-β), interleukin-10 (IL-10) to cause effector T cell anergy. In addition, they express CTLA-4 as another weapon of T cell inhibition [21]. Although ipilimumab antitumor effects are not completely understood, it was found that the CTLA-4 blockade enables enhanced T cell priming by more efficient antigen presentation and reduced T_reg_ function, all of which leads to more abundant effector T cell infiltration in the tumor microenvironment (TME) [16].

A second checkpoint protein, PD-1, also plays a role in the maintenance of self-tolerance. However, unlike CTLA-4, it becomes expressed in the effector phase of the immune reaction [16,22]. Anti-PD-1 axis monoclonal antibodies approved for melanoma treatment include nivolumab (anti-PD-1) [6] and pembrolizumab (anti-PD-L1) [5]. PD-1 is also termed as a marker of T cell exhaustion because T cells express it after prolonged or intense antigen stimulation, which in turn causes their suppression. Malignant tumors produce tumor neoantigens that are continuous stimulatory signals for T cells. The notion that a higher mutational burden in tumors leads to more potent T cell activation is supported by the finding that it is associated with a higher load of tumor infiltrating lymphocytes (TIL) in TME [23] and also with higher expression of markers of cytotoxicity in TME [24]. Although PD-1 is frequently described as an “exhaustion marker”, the accuracy of this term is unclear because it was shown that even during prolonged tumor antigen stimulation, some T cells preserve part of their functionality [21]. The PD-1-induced inhibitory signal triggers after its binding with PD-L1. PD-L1 can be expressed on a variety of cells. Melanoma, non-small-cell lung carcinoma (NSCLC) and ovarian cancer are tumors which highly express PD-L1, which is also a marker of worse clinical outcome [16]. ICI therapy has become especially important in the treatment of NSCLC, with a number of approved drugs, and others currently in trial or awaiting approval. PD-L1 expression seems to be a more relevant biomarker of ICI response in lung adenocarcinoma than in squamous cell lung cancer [25]. PD-1 axis activation has several implications depending on the cell type PD-1 is expressed on. It directs naïve T cell maturation towards the T_reg_ phenotype and helps in maintaining their immunosuppressive activity in the TME [16]. It also causes exhaustion of activated CD8+ cells, thus shielding tumor cells from their cytotoxic effect and allowing tumor cell control over the host immune system. It was found that dendritic cells (DCs) express PD-L1 following antigen binding. This is a protective mechanism against cytotoxic CD8+ cells, but also impairs their action in TME. By negative feedback, PD-L1 expression on DCs is upregulated by IFN-γ released from cytotoxic CD8+ cells [26]. 

Importantly, although TME can be rich in TILs, their function is affected by the chronic influence of tumor cell cytokines and chemokines and they become dysfunctional [21].

**Figure 1:**
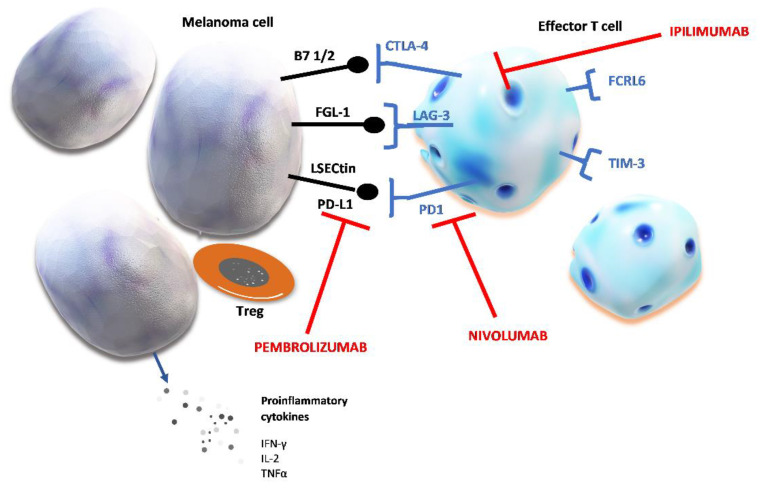
Chronic stimulation of T cells and markers of T cell exhaustion. Prolonged tumor neoantigen stimulation causes the upregulation of several inhibitory proteins. Checkpoint proteins expressed on effector T cells are: cytotoxic T-lymphocyte–associated antigen 4 (CTLA-4), lymphocyte activation gene 3 (LAG-3), programmed cell death protein 1 (PD-1), Fc receptor-like 6 protein (FCRL6), T cell immunoglobulin and mucin domain-containing protein 3 (TIM-3). Their ligands can be expressed on melanoma cell and after binding to their specific receptor, these ligands induce T cell exhaustion which shields tumor cells from host’s immune defense. The figure shows the specific ligands of immune checkpoint proteins: fibrinogen-like protein (FGL-1); liver sinusoidal endothelial cell lectin (LSECtin); programmed death-ligand 1 (PD-L1); peripheral membrane protein found on antigen presenting cells type 1 or 2 (B7 1/2).

Given its inhibitory effect on adoptive immunity, PD-1 and PD-L1 blockade causes reversal of these immunosuppressive effects. Figure 1 shows PD-1 and other markers of T cell exhaustion that are expressed on their surface upon chronic stimulation with tumor neoantigens.

Table 1 summarizes immune check point proteins with their specific ligands and mechanisms of their immunosuppressive effects. Altogether, their activation by the tumor results in creating perfect conditions for tumor cell proliferation and growth by suppressing host immune defense.

As mentioned earlier, ICI achieve remarkable therapeutic effects in a significant proportion of patients with advanced melanoma. It has been reported that certain characteristics of melanoma cells can be predictive of a good response to ICI. This primarily refers to the mutational load in malignant cells and expression of MHC molecules on their surface [27]. Melanoma and NSCLC [27] are known to have a high mutational burden, which is the rationale for the widespread use of ICI in their treatment [4,28,29]. Next, we discuss the known mechanisms of primary and acquired resistance to ICI in melanoma and explore future directions in the field of treatment of melanoma. 

## 3. Primary Resistance Mechanisms 

Primary resistance to PD-1-based immunotherapy manifests in 40–65% [38,39] and with ipilimumab therapy in more than 70% of melanoma patients [40]. To understand primary resistance mechanisms, we will first review the cancer immune cycle with an emphasis on important points for immunotherapy resistance.

When a malignant cell dies, it releases cancer neoantigens into the TME. Cancer neoantigens are recognized by the APC and bind to the MHC I molecule on the APC cell surface [41]. Dendritic cells are the major skin APCs that play a role in the defense mechanism against melanoma [42,43]. After binding the neoantigen, APC migrates to the tumor-draining lymph nodes. There, APC presents the tumor neoantigen to the naïve T cells, which causes them to transform into effector T cells, CD4^+^ or CD8^+^ lymphocytes. Subsequently, effector T cells enter the circulation and extravasate at the tumor site to infiltrate it and destroy the malignant cells. Cancer cell death is mediated by the cytotoxic CD8+ T cells via their granzymes and perforins release [41]. An overview of primary resistance mechanisms to ICI therapy is summarized in Table 2.

Factors that can impact on the first step in the cycle are the tumor mutational burden (TMB) and tumor immunogenicity. Proper APC/DC functioning and T cell priming processes are crucial in the next step in the cycle and any deviation from it can enable tumor cell immune evasion. In the final phase, T cell migration through the endothelium and tumor niche infiltration can be affected in several ways. T cell cytotoxicity can be attenuated by several mechanisms and this can also cause uncontrolled malignant cell proliferation [41]. An immunosuppressed environment results in escape mechanisms for malignant cells, enabling tumor progression. Given that currently available ICIs reverse the tumor inhibitory signals toward effector T cells, all the factors that dampen T cells at any stage of the antitumor immune response cause ICI therapy to be less effective. We discuss below each of the steps of the immune reaction against malignant cells and accentuate mechanisms of primary resistance to ICI therapy.

**Tumor mutational burden (TMB)** is defined as the number of nonsynonymous somatic mutations per genome megabase [44]. Tumors with a low mutational burden lack tumor-associated antigens (TAA), which would drive T cell priming [45]. Depending on TMB, antigens from TME can be tumor-associated antigens or shared antigens—the ones similar with normal antigens from the host. Both antigens are picked up by APCs. However, only the tumor-associated antigen presentation will result in T cell priming and strong tumor infiltration by the activated lymphocytes [46]. Tumors with a low mutational burden are poorly immunogenic and therefore can easily deceive the host’s antitumor immunity, which is mediated by T_reg_ activation [47]. Melanoma is characterized by high TMB, as are some other types of solid tumors that result from certain environmental exposure [48]. Higher TMB has been shown to be a positive prognostic factor across many tumor types treated with ICI. In one study of 10 different malignant tumors which were treated with one ICI or a combination of ICI, TMB positively correlated with overall survival [49]. TMB also positively correlated with PD-L1 status and a low mutational burden has therefore been associated with a poor response to PD-1-directed therapy [50,51]. In a literature review by Yarchoan M et al., it was reported that TMB across 27 different tumor types significantly correlated with a positive response to PD-1-directed immunotherapy in patients in whom PD-L1 expression was not analyzed [52]. TMB estimation has been recognized to be an important clinical test, and there has been an initiative to standardize the methods and panels used for this purpose [53]. A recently published study proposed guidelines for the use of different TMB estimation panels in 14 tumor types including melanoma. This was necessary because of biases that resulted from next generation sequencing panels that analyzed different size exomes and were inappropriately used across different cancer types missing a variability in their mutated genes [54]. It should be kept in mind, however, that the predictive value of TMB has been shown to be applicable in tumors in which the neoantigen load was proportional with cytotoxic CD8+ levels in TME, implying that this is not a unique predictive biomarker of ICI efficacy across all tumor types [55]. With regards to strategies for increasing TMB, radiotherapy is an option. However, conventional radiotherapy does not seem to cause a high enough increase in TMB to impact ICI efficacy [56]. Nevertheless, it has been noted that radiotherapy does increase the immunotherapy efficacy to some extent, which implies that mechanisms other than TMB increase are likely involved [57].

**Antigen presentation and T cell priming**. Dendritic cells (DC) are the key cells in triggering either immune response or tolerance [63]. Inappropriate response to antigen, therefore, leads to autoimmunity, chronic inflammation or malignancy. They act as professional APCs and after trafficking from TME into tumor-draining lymph nodes, they recruit T cells into effector CD8^+^ cells [64]. However, before this happens, DCs undergo the process of maturation which makes them efficient T cell inducers [65]. Proper DC maturation is crucial because the abundance of effector T cells in TME of melanoma directly depends on the density of DC [66]. In essence, any factor that impairs DC maturation or function also contributes to resistance to ICI. It was observed, for instance, that lipid accumulation in DC leads to their attenuated response to tumor antigens [67]. Tumor cells are capable of impairing DC maturation by maintaining the IL-6 and IL-10 levels in TME and can also direct their maturation into regulatory phenotypes [68]. Regulatory DCs (DC_reg_) can also arise from tumor cell influence mediated by arginase I (ARG1). DC_reg_ attenuate T cell response against tumors [69]. IL-35 was also identified as a suppressor of DC maturation [58], which is secreted by regulatory T and B cells, and also by melanoma cells [70,71]. 

**T cell trafficking and TME infiltration**. Effector T cell homing has been found to be a major determinant of not only favorable prognosis, but also of reactivity to immunotherapy in melanoma [72]. Studies have confirmed that the density of TILs and not circulating effector T cells is the prerequisite for a response to immune checkpoint inhibition and other immunotherapy strategies [73]. C-X-C Motif Chemokine Receptor 3 (CXCR3) is a G protein-coupled chemokine receptor in effector T cell membrane. Its ligand expression on DC is upregulated by IFN-γ and CXCR3 engagement with its ligands is an important step in effector T cell homing [74]. The study by Chow et al., in a mouse model, demonstrated that the interaction between effector CD8+ cells and CD103+ DCs via CXCR3-CXCL9 complex is a prerequisite for efficient anti-PD-1 therapy. It also suggested that this interaction restored the cytotoxicity of CD8+ cells that were already present in TME [59]. CXCR3 upregulation, therefore, is a potential new avenue for research in melanoma treatment that may increase the sensitivity to ICI therapy [60,75].

**TME infiltration**. Effector T cell infiltration is a predictor of response to ICI therapy. Vascular endothelial growth factor (VEGF) is an important mediator in tumor angiogenesis. However, besides neovascularization, it also has an inhibitory effect on effector T cell homing by disabling their trafficking across endothelium [76]. Under the influence of VEGF and other mediators, new tumor vessels of an irregular structure arise. Apart from their effect on tumor angiogenesis, they also stimulate immunosuppressive microenvironment and disable T cell infiltration [77,78]. VEGF compromises T cell trafficking by downregulation of proteins necessary for their passage through the endothelial wall: vascular cell adhesion protein 1 (VCAM-1) and integrin ligands intercellular adhesion molecule 1 (ICAM-1) on endothelium [41]. Blood vessels within tumors differ from normal vessels in morphology and function, which dampens T cell trafficking across endothelium. Anti-VEGF therapy leads to the transformation of such tumor blood vessels into normal ones, also termed vascular normalization, and thereby restores T cell trafficking [79]. However, this is a transient effect. Studies have shown that another mediator of angiogenesis, angiopoietin 2 (ANG2), in circumstances when VEGF is inhibited, can actually maintain abnormal tumor angiogenesis and thereby overcome anti-VEGF therapy effects [80]. Based on these characterizations of intrinsic resistance to immunotherapy, there have been some preclinical and early clinical results that proposed the addition of anti-VEGF therapy [76,81,82,83,84] or dual anti-VEGF/anti-ANG2 therapies to ICI therapy to improve efficacy. More clinical trials are needed [61]. 

**Figure 2:**
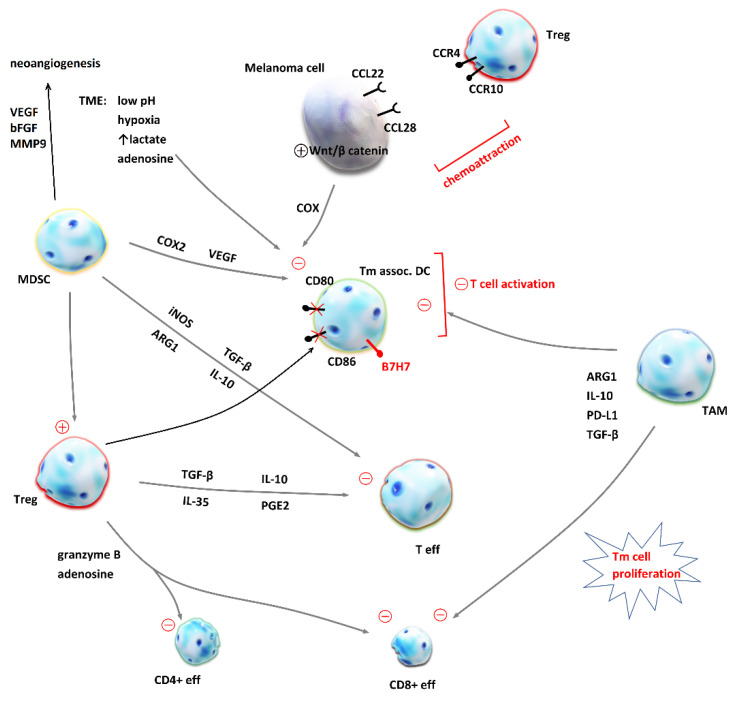
Mechanisms of immune suppression in the tumor microenvironment (TME). Malignant cells evade host’s antitumor response by the chemoattraction of immunosuppressive cells into the TME and the creation of a milieu which inhibits effector T cells (T_eff_). Regulatory T cells (T_reg_) release transforming growth factor β (TGF-β), interleukin-10 (IL-10), interleukin-35 (IL-35) and prostanglandin E2 (PGE2), which inhibit T_eff_ proliferation and cytokine release. In addition, T_reg_ secrete granzyme B and adenosine [87] and lead to apoptosis of effector CD4+ and CD8+ T cells [88]. Cytotoxic CD8+ T cells are also inhibited by the tumor-associated macrophages (TAM) as they produce arginase 1 (ARG1) [89], IL-10, programmed death-ligand 1 (PD-L1) and TGF-β [90]. Dendritic cells (DC) are crucial for T cell priming and their inhibition by TAM, myeloid-derived suppressor cells (MDSC), melanoma cells and unfavorable conditions in the TME contribute to immunosuppression and resistance to immune checkpoint inhibition. MDSC produce inducible nitric oxide synthase (iNOS), TGF- β, ARG1 and IL-10, which inhibit effector T cells. On the other hand, MDSC inhibitory effect on DC is mediated by cyclooxygenase 2 (COX2) and vascular endothelial growth factor (VEGF) [91,92]. MDSC are also crucial for neoangiogenesis and tumor invasiveness, because they secrete VEGF, matrix metalloproteinase 9 (MMP9) and basic fibroblast growth factors (bFBF) [93]. Under the influence of T_reg_, DC express co-inhibitory membrane protein B7H7 and CD80 and CD86 are downregulated [90]. This impairs T cell activation. Chemoattraction of T_reg_ is achieved by the expression of CCL22 and CCL28 on melanoma cell, ligands for CCR4 and CCR10 expressed on T_reg_ [90]. The activation of an intrinsic Wnt/β-catenin pathway in melanoma cell has been linked to deprived T cell infiltration of TME [92].

**Immunosuppressive TME**. Tumor cells are capable of creating an environment that promotes their vitality and tumor expansion. They deceive the host immune system by controlling its protective mechanisms. Immune cells involved in tumor tolerance are regulatory T cells (T_reg_), tumor-associated macrophages (TAM) and myeloid-derived suppressor cells (MDSC), which are stimulated and APCs and effector T cells, which are suppressed. In addition, TME rich in lactate and adenosine, with low pH and hypoxia, also have an inhibitory effect on APCs. Such control over the host immune response is mediated by several key factors: transforming growth factor β (TGF-β), VEGF, IL-10, PGE2, ARG1, and IL-35 [61,85]. More details about the mechanisms of immune suppression in the tumor microenvironment are depicted in Figure 2. Regulatory T cells have a role in maintaining tolerance towards the host tissues by suppressing an exaggerated immune response. Tumor cells use them to suppress every stage of T cell activation and effector T cell function in TME. The ratio between effector CD8+ cells and T_reg_ in TME is found to positively correlate with prognosis in melanoma patients, which offered a basis for T_reg_ depletion strategies to evolve as potential contributors to ICI therapy [62,86].

A different mechanism of intrinsic resistance to ICI therapy associated with host characteristics is the composition of the gut microbiome. Gut microbiome composition was observed to differ between responders and non-responders to anti-PD-1-targeted therapy and may serve as an additional predictor of response to ICI therapy [94]. Preclinical and clinical studies on a small number of patients have shown some benefit of fecal microbiota transplantation in improving ICI therapy efficacy [95,96,97].

## 4. Mechanism of Acquired Resistance

Acquired resistance develops after a period of clinical response to treatment following which the tumor progression occurs. There are certain changes observed in ICI-resistant melanoma cells that are postulated to play role in acquired resistance to ICI and are discussed below. It should be kept in mind that there are many resistance mechanisms which overlap between primary and acquired types.

Changes that lead to acquired resistance can be developed at the cell population or at single cell levels. Acquired resistance that occurs at the cell population level includes selection of a cell subpopulation that possesses required characteristics not only to overcome the treatment, but also to grow and substitute the initially present cell population sensitive to anti-cancer therapy. When a single malignant cell is observed, changes in its biology occur in response to interaction with the host’s immune system or cancer therapy and enable their escape from either of them [98].

**Figure 3:**
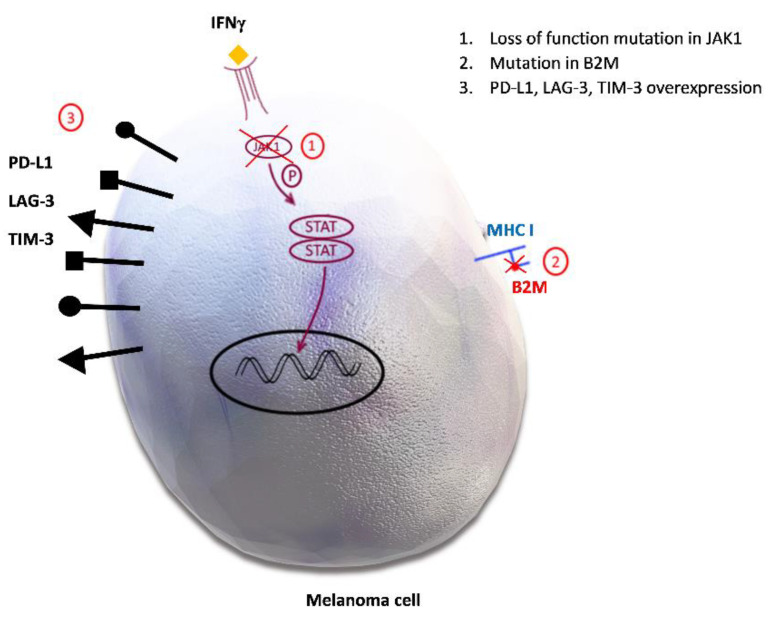
Potential mechanisms of acquired resistance to immune checkpoint inhibitors. 1. Loss of function mutation in Janus kinase 1/Signal transducer and activator of transcription (JAK1/STAT) inhibits downstream signal transduction and the interferon gamma (IFN-γ) effect on melanoma cells. 2. Mutation in beta 2 microglobulin (B2M) causes defective B2M and major histocompatibility complex I (MHC I) molecule, therefore disabling the priming of cytotoxic CD8+ cells. 3. Programmed death-ligand 1 (PD-L1), lymphocyte activation gene-3 (LAG-3), T-cell immunoglobulin and mucin domain 3 (TIM-3) overexpression promotes inhibitory signals in effector T cells, leading to their anergy.

We discussed earlier how impaired antigen presentation contributes to primary resistance to ICI by reduced T cell priming. Deficient antigen presentation also plays a role in acquired drug resistance. Loss of **beta 2 microglobulin (B2M),** a component of MHC class I molecules which is involved in priming of CD8+ cells, results in an impaired process of regulation of cytotoxic T lymphocytes (CTL) [9,99,100]. It has been observed that B2M mutations associated with the loss of MHC I expression are present in patients who developed acquired resistance to ICI [99]. The B2M-positive tissues of patients before the ICI treatment and an absence of B2M in lesions of patients with melanoma progression implies that the loss of B2M is responsible for acquired resistance [9,99,100]. Figure 3 shows B2M loss and some other markers of acquired resistance to ICI therapy.

**Janus Kinase 1 and 2/Signal transducer and activator of transcription) (JAK 1, 2/STAT) signaling pathway** is involved in many different biological processes, including cell differentiation, growth, apoptosis, and migration and plays a very important role in patient immune system [101]. Genetic changes in JAK/STAT pathway have been reported in selected cases of pembrolizumab-resistant melanoma cells. IFN-γ excreted from T cells binds to tumor cells and recruits JAK1/JAK2. This subsequently leads to migration of the activated STAT1 (transcription factor) into the nucleus and transcription of the IFN-γ-inducible genes. In this way, tumor cell growth is stopped and tumor cell apoptosis, as well as T cell infiltration, are induced [10]. Loss of function mutations in JAK1, therefore, disable this mechanism and contribute to PD-1 inhibitor resistance [9,100]. 

It was noted that melanoma develops resistance by overexpression of ligands to immune checkpoints, and also to alternative inhibitory proteins, which contributes to further melanoma growth [34]. **PD-L1** **expression** is a tumor defense mechanism which leads to cytotoxic CD8+ T cell exhaustion and contributes to tumor immune evasion by shifting the maturation of naïve CD4+ cells into T_reg_. PD-L1 upregulates as a response to IFN-γ [9]. There are several alternative immune checkpoints identified which have an impact on immunosuppression in melanoma and some other solid malignancies. They are listed in Table 1. 

**Lymphocyte activation gene 3** (LAG-3) expressed on the T cell surface has also been associated with the acquired resistance to immunotherapy [9]. LAG-3 is the inhibitory immune checkpoint receptor, also known as CD223, that negatively regulates CD8 and CD4 T cells and its soluble form reduces the differentiation from monocytes to DCs and macrophages, which causes the appearance of new APCs with reduced immunostimulatory function [33]. Because LAG-3 has an impact on cell proliferation, cytokine secretion, immune system defense mechanism and homeostasis, it plays an important regulatory role in the immune system [102,103]. In addition to the cell surface, LAG-3 protein can be also present in lysosomes, which enables it to move fast on the cell surface upon T cell activation. Bae et al. demonstrated that degradation of LAG-3 in lysosomes plays a major role in the prevention of LAG-3 expression on the cell surface. Therefore, translocation of LAG-3 to the cell surface would be enabled by the inhibition of the lysosomal degradation process. LAG-3 trafficking to the cell surface upon its stimulation occurs by the cytoplasmic domain of LAG-3 via the protein kinase signaling pathway, whereby protein kinase C (PKC) does not modify its cytoplasmic domain [104]. Expression of LAG-3 is triggered by TCR or cytokine stimulation [103] and it interacts as a ligand of the MHC II molecule expressed on APCs and tumor cells [33]. Similar to PD-1 and CTLA-4, this membrane protein upon activation dampens the autoimmunity in circumstances of prolonged antigen stimulation. It reduces T cell proliferation and potentiates T_reg_ function and in those ways contributes to immune evasion [33]. Tumor infiltrating T cells constantly exposed to the antigen, express LAG-3 and also multiple other co-receptors, which results in their exhaustion [105]. Indeed, LAG-3 showed a similar activity to other known checkpoint proteins and is co-expressed with PD-L1, TIM-3 and T cell immunoreceptor with immunoglobulin and ITIM domain (TIGIT). Co-inhibition of PD-1 and LAG-3 improved cytotoxic T cell function in experimental models [106].

Another transmembrane protein, **T-cell immunoglobulin and mucin domain 3 (TIM-3),** showed the same effect on host defense mechanisms and is thought to contribute to acquired resistance [9]. Studies have shown that TIM-3 overexpression is related to T cell exhaustion and poor prognosis in cancer patients [33]. TIM-3 was discovered on the surface of many different cells such as CD4+ cells, CD8+ cells, T_reg_, NK cells and DCs and in TME it binds to four different ligands (Gal-9, HMGB1, Phosphatidylserine, and Ceacam-1). When those ligands are not available, adaptor molecule Bat3 binds to the cytoplasmic tail of TIM-3 in order to avoid intracellular inhibitory signaling and cell death or T cell exhaustion. Engagement of TIM-3 with these ligands leads to phosphorylation of the cytoplasmic tail and to its disconnection from Bat3 [107,108]. TIM-3 has a key function in cytokine expression and in blocking T helper 1 (Th1) responses and was originally identified as a marker for CD4+ Th1 and CD8+ T cytotoxic 1 cells, which are producing IFN-γ [107,109]. TIM-3 dysfunction has been linked to autoimmune diseases such as multiple sclerosis, rheumatoid arthritis and diabetes mellitus type 1 [108,109]. In metastatic melanoma, expression of TIM-3 is upregulated in CD4+ and CD8+ TILs, which leads to CD8+ T cells exhaustion, which are enabled to produce TNF-α, IFN-γ or IL-2 [108,110,111]. Poor cancer prognosis is related to TILs, which express both TIM-3 and PD-1 and therefore combination therapy with anti-TIM-3 and anti- PD-1 could contribute to more effective tumor inhibition [108]. Indeed, while a single TIM-3 blockade has demonstrated a not very significant effect, dual TIM-3 and PD-1 blockade was more successful in achieving an antitumor response [110]. 

It is very likely that there is a much higher number of inhibitory proteins and their ligands that play roles in tumor expansion and resistance development. One of the recently identified and studied is **Fc receptor-like 6 protein (FCRL6)**, which belongs to the immunoglobulin super family (IgSF). There are six members including IgSF and FCRL1–5, which are important in B cell function, while FCRL6 is involved in T cell function [112]. FCRL6 is a membrane inhibitory protein expressed on mature cytotoxic NK and T cells, which interact with other cells by binding major histocompatibility complex molecule/human leucocyte antigen – DR isotype (MHC II/HLA-DR) on their cell surface [11]. Its engagement with ligand activates the axis which is important in antigen presentation and cytotoxicity in T cells [112]. Importantly, MHC II presence in tumors is a predictor of good sensitivity for anti-PD-1 therapy. However, with chronic anti-PD-1 therapy of MHC II^+^ tumors, FCRL6 becomes overexpressed, leading to the disruption of antitumor immunity in a distinctive manner [37]. Consequently, activation of the FCRL6-MHC II axis leads to immunosuppression by downregulation of cytotoxic NK cells and effector T cells and results in acquired resistance to anti-PD-1 treatment. Apart from FCRL6 and MHC II, LAG-3 is also found to be overexpressed and plays a role in this particular mechanism of immune evasion of melanoma, triple negative breast cancer, and non-small cell lung cancer cells [37].

## 5. Future Directions and Conclusion

### 5.1. New Drugs Currently in Clinical Trials

Simultaneous inhibition of two or more immune checkpoints is the logical next step in therapeutic strategy. Combination therapy with a CTLA-4 inhibitor and a PD-1 inhibitor has already been approved, i.e., ipilimumab and nivolumab [8,99]. Another strategy is the combination of radiotherapy and PD-1/PD-L1 inhibitor therapy, due to its effect of increasing effector T cell infiltration of tumor and PD-L1 expression, therefore making it more sensitive to immunotherapy. This principle is worth exploring further and will require close radiotherapy regimen tuning to produce optimal effects in combination with ICI therapy [57]. 

Another avenue worth exploring is the modification of the gut microbiome. Preclinical studies have indicated that gut microbiome has an immunomodulatory effect [113]. For example, colonization with *Bifidobacterium longum, Collinsella aerofaciens* and *Enterococcus faecium* was more abundant in responders to anti-PD-1 therapy. The same study, conducted on a mouse model, found that responders had TME rich in CD8+ T cells and poor in T_reg_. The findings were opposite in non-responders [94]. Literature data also suggest that certain commensal gut bacteria were associated with the reduction of T_reg_ and MDSC in the circulation [114]. Several studies have shown that non-responders’ gut microbiome was characterized by low diversity and abundance in *Bacteroidales* order [96,114]. These observations were the rationale for designing the trials of fecal microbiota transfer as a therapeutic option to enhance tumor response to ICI. In preclinical studies, it was shown to be promising [94]. Recently, a published clinical study reported success in reverting anti-PD-1 resistance in 6 out of 15 patients to whom respondent fecal microbiota transplantation was performed, linking the change in gut microbiota with response to anti-PD-1 therapy [97].

Due to the definite benefit that ICI has brought to melanoma treatment, and especially given that the combination of two ICIs showed improved results compared to single agents, new immune checkpoints are being explored. These potential new drugs may further improve outcomes alone or in combination with currently available agents. However, the challenge of avoiding severe toxicity observed in drug combinations remains to be tackled. One such candidate is LAG3 [34]. Clinical trials on melanoma patients with agents affecting this checkpoint protein are already under way (NCT01308294, NCT02676869, NCT00365937, NCT00324623). Another potential mechanism to be targeted is TIM-3 [34]. Details about current trials investigating the efficacy of anti-TIM-3 agents in melanoma can be found by searching these trials: NCT04139902, NCT02817633 and NCT02791334. Clinical trial investigating the effect of vibostolimab, an antibody to TIGIT in melanoma, is also under way (NCT04303169) and is given together with other ICIs. 

Elevated concentrations of adenosine within the TME have also been identified as potential immune escape mechanisms. ATP is mostly an intracellular molecule and its extracellular concentration increases only under pathophysiological conditions, such as TME, in order to boost immune system response. Enzyme CD39 converts extracellular ATP/ADP into AMP, while in the last irreversible step, enzyme CD73 converts AMP into ADO, which binds to A1R, A2AR, A2BR, and A3R receptors on the effector T, NK, tumor cell and tumor associated macrophages (TAMs) [115]. CD39 and CD37 are both expressed on infiltrating immune cells and CD39 is also expressed on melanoma cells and T_reg_ [116,117]. In contrast to ATP, ADP dampens immune surveillance and causes melanoma progression [115]. Blocking this adenosinergic axis could have an important therapeutic effect and, therefore, drugs that target these enzymes and A2AR and A2BR have already reached clinical trials [118]. 

### 5.2. Mechanisms that Hold Promise for Potential Interventions to Limit Metastatic Melanoma

Protein tyrosine phosphatase 2 (PTPN2) dephosphorilates STAT1 and JAK1 and are thereby involved in modulation of IFN-γ signaling. By *in vivo* clustered regularly interspaced short palindromic repeats (CRISPR) screening, Manguso et al. identified that *PTPN2* deletion led to enhanced IFN-γ signaling and antigen presentation. In addition, it potentiated tumor cell apoptosis, which altogether markedly increased the response to immunotherapy [119].

Adenosine deaminase acting on RNA 1 (ADAR1) is an enzyme which edits double stranded RNA (dsRNA) by deamination of adenosine to inosine (A to I editing) [120] and was discovered to act as a checkpoint by inhibiting the sensing of endogenous dsRNA [121], which accumulates in pathological circumstances [120]. Three different studies demonstrated that loss of function mutation in the *ADAR1* gene overcomes resistance to PD-1 blockade in tumors which express IFN- stimulated genes [122,123,124]. This was based on the finding that the addition of IFN to ADAR1-deficient tumor cell culture led to a reduction in tumor cell viability. Antitumor immune reaction and tumor growth arrest are mediated by dsRNA immune sensors melanoma differentiation-associated protein 5 and dsRNA-activated protein kinaze R, respectively [125]. Ishizuka et al. also propose that anti-PD-1 therapy can be effective without the presence of activated tumor-specific CD8+ T cells when ADAR1 is inhibited, because it produces significant inflammation and reduces tumor size [122]. 

Both these mechanisms emerged as another potential drug targets for increasing anti-PD-1 therapy efficiency.

A different focus was targeted by Imbert et al. in their study which revealed how sfingosine kinaze 1 (SK1) contributes to melanoma immune evasion by T_reg_ accumulation and few other immunosuppressive changes in TME [126]. SK1 is involved in ceramide metabolism and is responsible for phosphorylation of sphingosine to sphingosine-1-phosphate (S1P). In melanoma and many other tumors, this enzyme is overexpressed, ultimately causing increased production of S1P. Various studies showed that SK1 overexpression was a poor prognostic predictor in different tumors [127]. S1P is an oncogenic mediator that acts by binding to G-coupled receptor and influences tumor cells, vascular endothelium and lymphocytes [128]. Its pro-oncogenic effect is based on anti-apoptotic and pro-proliferative effect on tumor cells. S1P was also found to be one of the crucial mediators in tumor metastasis in various tumors [129]. Its action on lymphocyte results in cytotoxic CD8+ T cell inhibition, while SK1 inhibition leads to decreased TME infiltration by T_reg_. Based on these findings from the literature, targeting SK1 could lead to improved patient outcomes by enhancing the response to ICI in patients with advanced melanoma [126].

Indoleamine 2,3-dioxygenase (IDO), an enzyme that converts tryptophan into kynurenine, is also overexpressed in melanoma. T cells require tryptophan for their function and, therefore, its depletion suppresses T cell function. It has been demonstrated that when a tryptophan analogue in mice was administrated, the tumor development was significantly slowed [9]. IDO appears to be a new potential target in the research of resistance to ICI therapy, and has, in combination with ICI, successfully passed the preclinical trials [9,99]. However, phase III clinical trials showed no benefit of IDO-inhibitors in combination with anti-PD-1 therapy [130]. Nevertheless, this pathway could still be exploited by targeting additional or different mediators [131]. The new possible target could be tryptophan 2,3-dioxygenase (TDO), the rate-limiting first enzyme of the kynurenine pathway in liver that also converts tryptophan into kynurenine and contributes to the suppression of the immune system [131,132,133]. Similar *in vitro* activity has shown few anti-TDO molecules, but only EOS200809 *in vivo* activity has been confirmed in this. Schramme et al. demonstrated that in tumors which do not express TDO, administration of anti-TDO (EOS200809) could help in overcoming IDO-induced immunosuppression by elevating concentrations of tryptophan [131]. Except IDO and TDO various enzymes of the kynurenine pathway have been identified as potential targets in cancer treatment such as nicotinamide phosphoribosyltransferase (NMPRT), nicotinamide N-methyltransferase (NNMT) and poly-(ADP-ribose) polymerase (PARP) [132].

Inhibition of histone deacetylase (HDAC) in combination with PD-1 inhibitors improved therapeutic response in comparison to the placebo group and anti-PD-1 monotherapy [9]. HDAC deacetylates histones and, therefore, has an impact on DNA transcription and expression of the genes involved in prolonged tumor cell survival and escape from immune surveillance [134]. However, reversible acetylation of many other proteins involved in tumor cell destruction, such as heat shock protein 90 and the p65 (subunit of nuclear factor kappa-light-chain-enhancer of activated B cells, NFκB ), is controlled by HDAC [135]. Elevated levels of HDAC and modified histones are present in advanced melanoma and contribute to its poor prognosis. Furthermore, melanoma cells under conditions of increased HDAC concentrations via PD-L1 activate MDSCs and these cells promote resistance to ICI by causing the exhaustion of CD8+ T cells [134]. Studies have shown that MDSCs are inactivated when HDAC inhibitor valproic acid, in combination with a PD-1 inhibitor, was administered [136]. Moreover, nexturastat A, a selective HDAC6 inhibitor, prevents increased expression of PD-L1 via anti-PD-1 antibodies [137]. These findings suggest that targeting HDAC in patients with resistant melanoma could be promising and could become standard melanoma therapy in combination with immune checkpoint inhibitors [134]. 

A somewhat different approach is the inhibition of proprotein convertase subtilisin/kexin type 9 (PCSK9). This protein is involved in cholesterol metabolism and monoclonal neutralizing antibodies evolocumab and alirocumab are now approved for hypercholesterolemia treatment. It is also of interest in melanoma therapy because it attenuates effector T cell homing into TME. One study showed that PCSK9 neutralization improved the efficacy of ICI in melanoma in a mouse model [138]. Other potential targets with information about ongoing trials can be found in the literature [34,139] (Table 2 in [99]) and their abundance offers hope that melanoma treatment will advance further.

From the data presented above, we can see that despite the revolution that ICI has caused for melanoma patients, successful treatment still faces a long road ahead. A combination of current and future drugs targeting different resistance mechanisms seems like a reasonable solution, but agents with lesser toxic effects are still needed.

## Figures and Tables

**Table 1:** Immune checkpoint proteins, their ligands and biological effect.

Immune Checkpoint (IC)	Cells Expressing IC	Upregulation Signal for IC	IC Ligands (L)	Cells Expressing Ligands	Effect of Engagement of IC-L	Reference
CTLA-4	CD8+ CD4+ T_reg_	CD28-B7 complex	B7-1 B7-2	APC	Pro-apoptotic effect on CD8+cells T cell exhaustion	[16,19,30]
PD-1	Effector T cells (T, B cells) APC NK cells	IFN-γ from effector T cells	PD-L1 PD-L2	TILs Treg Macrophages DCs Tumor cells	Pro-apoptotic effect on CD8+ cells T cell exhaustion	[9,16,30]
LAG-3	Activated T cells (CD4+, CD8+) Subset of NK cells T_reg_ TILs Tumor cells	Chronic stimulation of effector T cells; IFN-γ, TNF-α	Galectin-3 LSECtin FGL-1 L-selectin MHC II/Ag complex	Tumor cells (melanoma) APCs Stromal cells (cancer-associated fibroblasts, myeloid-derived suppressor cells)	Reduction in CD4+ cells proliferation (T cell exhaustion) Upregulation of T_reg_ Inhibition of DC maturation	[31,32,33]
TIM-3	Activated T cells DCs T_reg_	Chronic stimulation of effector T cells: TGF-β	Galactin-9 CAECAM1 HMGB1 Phosphatidyl serine	APC Tumor cell	Dampening CD8+ recruitment Dysfunctional CD8+ cells (T cell exhaustion) Tumor progression	[34,35,36]
FCRL6	Mature NK cells Activated T cells	Chronic anti-PD therapy in MCH II^+^ tumor	MHC II/HLA-DR	Tumor cells MHC II^+^ (melanoma, Hodgkin’s disease, breast and ovarian cancer)	NK cell suppression T_eff_ suppression (T cell exhaustion)	[11,37]

CTLA-4—cytotoxic T-lymphocyte–associated antigen 4; APC—antigen presenting cell; PD-1—programmed cell death protein 1; TILs—tumor-infiltrating lymphocytes; DCs—dendritic cells; LAG-3; LSECtin—liver sinusoidal endothelial cell lectin; FGL-1—fibrinogen-like protein 1; TIM-3—T cell immunoglobulin and mucin domain-containing protein 3; CAECAM1—carcinoembyronic antigen-related cell adhesion molecule 1; HMGB1—high mobility group protein B1; FCRL6—Fc receptor-like 6 protein; MHC II—major histocompatibility complex molecule; HLA-DR—human leucocyte antigen—DR isotype.

**Table 2:** Mechanisms of primary resistance to ICI treatment.

Melanoma Characteristics/ Tumor Immune Cycle Stage	Mechanism of Primary Resistance	Strategies to Overcome Resistance Mechanisms	Reference
1. Tumor imunogenity	Low TMB Antigen loss	CTLA-4 inhibition Radiotherapy + ICI ?	[16,19,30,57]
2. Antigen presentation and T cell priming	Impaired DC maturation (IL-6, IL-10 from TME; lipid accumulation; IL-35)	Anti-IL-35 Ig ?	[58]
3. T cell trafficking and TME infiltration by TILs	VEGF overexpression ANG2 overexpression	Combination anti-VEGF + ICI Combination anti-VEGF + anti-ANG2 CXCR3 upregulation	[59,60,61]
4. T cell cytotoxic effect on tm cell	TIL inhibition by T_reg_	Treg suppression: sunitinib Cytotoxic T cell therapy	[62]

TMB—tumor mutational burden; CTLA-4—cytotoxic T lymphocyte associated antigen 4; ICI—immune checkpoint inhibition; DC—dendritic cell: TME—tumor microenvironment; VEGF—vascular endothelial growth factor; ANG—angiopoietin 2; ICI—immune checkpoint inhibition; CXCR3—chemokine receptor 3; TIL—tumor infiltrating lymphocytes; Treg—regulatory T cells.

## Data Availability

Data sharing is not applicable to this article as no new data were created or analyzed in this study.

## Abbreviations

APCs	Antigen-presenting cells
ANG2	Angiopoietin 2
Bat3	HLA-B-associated transcript 3
B2M	Beta 2 microglobulin
Ceacam-1	Carcinoembyronic antigen-related cell adhesion molecule-1
CTL	Cytotoxic T lymphocytes
CTLA-4	Cytotoxic T-lymphocyte-associated protein 4
CXCR3	C-X-C Motif Chemokine Receptor 3
CXCL9	C-X-C Motif Chemokine Ligand 9
DCs	Dendritic cells
DC_reg_	Regulatory dendritic cell
FCRL6	Fc receptor-like 6 (protein)
FGL-1	Fibrinogen-like protein 1
Gal-9	Galectin-9
HDAC	Histone deacetylase
HMGB1	High mobility group protein B1
ICI	Immune checkpoint inhibitors
IDO	Indoleamine 2,3-dioxygenase
JAK 1, 2	Janus Kinase 1, 2
LAG-3	Lymphocyte activation gene 3
LSECtin	Liver sinusoidal endothelial cell lectin
MDSCs	Myeloid-derived suppressor cells
NK cells	Natural killer cells
PD-L1/2	Programmed death-ligand 1/2
PD-1	Programmed cell death protein 1
PCSK9	Subtilisin/kexin type 9
STAT	Signal transducer and activator of transcription
SK1	Sfingosine kinaze 1
S1P	Sphingosine-1-phosphate
TCR	T-cell receptor
TDO	Tryptophan 2,3-dioxygenase
TIGIT	T-cell immunoreceptor with immunoglobulin and ITIM domain
TIL	Tumor infiltrating lymphocytes
TIM-3	T cell immunoglobulin and mucin-domain containing molecule-3
TMB	Tumor mutational burden
TME	Tumor microenvironment
T_reg_	T regulatory cells
VEGF	Vascular endothelial growth factor
